# Prediction of lung overdistension during mechanical ventilation using micro-RNA and gene expression

**DOI:** 10.1186/s40635-025-00768-2

**Published:** 2025-06-07

**Authors:** Cecilia López-Martínez, Paula Martín-Vicente, Laura Amado-Rodríguez, Inés López-Alonso, Margarita Fernández-Rodríguez, Adrián González-López, Pablo Martínez-Camblor, Juan Gómez, Andrew J. Boyle, Cecilia M. O’Kane, Daniel F. McAuley, James N. Tsoporis, Claudia dos Santos, Guillermo M. Albaiceta

**Affiliations:** 1 Centro de Investigación Biomédica en Red (CIBER)-Enfermedades Respiratorias, Instituto de Salud Carlos III, Madrid, Spain; 2 Instituto de Investigación Sanitaria del Principado de Asturias, Oviedo, Spain; 3 Unidad de Cuidados Intensivos Cardiológicos, Hospital Universitario Central de Asturias, Avenida de Roma S/N, 33011 Oviedo, Spain; 4 Departamento de Biología Funcional, Instituto Universitario de Oncología del Principado de Asturias, Universidad de Oviedo, Oviedo, Spain; 5 Department of Anesthesiology and Operative Intensive Care Medicine CCM/CVK, Charité-Universitätsmedizin Berlin, Corporate Member of Freie Universität Berlin, Humboldt Universität zu Berlin, and Berlin Institute of Health, Berlin, Germany; 6 Department of Biomedical Data Sciences, Geisel School of Medicine, Dartmouth College, Hanover, USA; 7 Redes de Investigación Cooperativa Orientadas a Resultados en Salud (RICORs), Madrid, Spain; 8 Laboratorio de Genética, Hospital Universitario Central Asturias, 33011 Oviedo, Spain; 9 Wellcome-Wolfson Institute for Experimental Medicine, School of Medicine Dentistry and Biomedical Science, Queen’s University, Belfast, UK; 10 Keenan Research Centre for Biomedical Science, St Michael’s Hospital, University of Toronto, 30 Bond Street, Room 4-008, Toronto, ON M5B 1WB Canada

**Keywords:** Lung stretch, Transcriptomics, Micro-RNAs, Overdistension

## Abstract

**Background:**

Overstretching of lung parenchyma may lead to injury, especially during mechanical ventilation. To date, there are no specific biomarkers of lung stretch, but transcriptomic signatures have not been explored. Our objective was to identify stretch-specific signatures using micro-RNA and gene expression.

**Methods:**

Data on micro-RNA and RNA expression in response to stretch in experimental models were systematically pooled. Signatures were identified as those micro-RNAs or genes with differential expression in samples from stretched cells or tissues, and optimized using a greedy algorithm. Expression data was used to calculate transcriptomic scores. The accuracy of these scores was validated in animal models of lung injury, ex vivo mechanically ventilated human lungs, and bronchoalveolar lavage fluid (BALF, *n* = 7) and in serum samples (*n* = 31) of mechanically ventilated patients.

**Results:**

Six micro-RNAs (mir-383, mir-877, mir-130b; mir-146b, mir-181b, and mir-26b) were differentially expressed in stretched cell cultures (*n* = 24). Amongst the genes regulated by these micro-RNAs, a 451-gene signature was identified in vitro (*n* = 106) and refined using data from animal models (*n* = 143) to obtain a 6-gene signature (Lims1, Atp6v1c1, Dedd, Bclb7, Ppp1r2 and F3). Transcriptomic scores were significantly higher in samples submitted to stretch or injurious mechanical ventilation. The microRNA and RNA signatures were validated in human tissue, BALF and serum, with areas under the ROC curve between 0.7 and 1 to identify lung overdistention.

**Conclusions:**

Lung cell stretch induces the expression of specific micro-RNA and genes. The potential of these signatures to identify lung stretch in a clinical setting must be explored.

**Supplementary Information:**

The online version contains supplementary material available at 10.1186/s40635-025-00768-2.

## Background

Lungs are exposed to mechanical stretch with each breath. Mechanical forces generated are sensed by the alveolar epithelium [1] and capillary endothelium [2] and, through a process of mechanotransduction, may trigger biological responses [3]. Excessive stretch and/or cyclic cellular deformation leads to abnormal responses including inflammation [4], matrix remodeling [5] and apoptosis [6]. Patients receiving mechanical ventilation may be exposed to injurious overdistension and repetitive cellular deformation that can have pathophysiological and clinical consequences, collectively termed as ventilator-induced lung injury (VILI) [7]. Avoidance or reduction of VILI is one of the main therapeutic goals in ventilated patients with respiratory failure.

Stretch is a continuous phenomenon with heterogeneous distribution along the parenchyma in both healthy and injured lungs, with alveolar areas submitted to variable amounts of deformation ranging from static conditions to 20% changes in size [8–10]. In vivo estimation of lung stretch remains a challenge. Imaging techniques can detect hyperaerated lung areas [11], but these may not reflect overstretching [12]. Assessment of stretch using lung mechanics during mechanical ventilation is based on high end-inspiratory or driving pressures, but these values may be influenced by an abnormal chest wall (causing an increase in airway pressures without overstretching) or large areas with high compliance that result in low pressures in spite of regional hyperinflation [13]. An alternative is to exploit the injurious effects of mechanical force by following biomarkers of lung injury [14]; however, no specific biochemical markers for overstretching have been identified to date. Characterization of a quantitative biomarker specific for stretch would make possible monitoring of lung distension even in global lung measurements such as bronchoalveolar lavage fluid (BALF), which are available in the clinical practice.

Several approaches using either metabolomics (35,925,420) or inflammatory mediators have been tested. Transcriptomic scores, based on quantification of several RNAs and their integration in a single value, have been proposed as a tool to overcome the complexity of syndromic diagnosis [15]. Several mRNA signatures for acute lung injury and VILI have been proposed [16], but their sensitivity to differentiate stretch from other forms of lung injury has not been tested, and have not been applied at the bedside, in part due to the paucity of lung tissue in which to perform measurements. Micro-RNAs (miRNAs) comprise a class of small, noncoding RNAs that control expression of complementary target mRNAs. Despite the existence of RNases, miRNAs remain stable in body fluids and detection of circulating miRNAs in serum/plasma suggests that miRNAs may fulfill biological functions outside the cell and serve as potential biomarkers for diseases. Circulating miRNAs are protected from RNase-dependent degradation by several mechanisms, including their inclusion in microvesicles, exosomes, and apoptotic bodies as well as through the formation of protein–miRNA complexes resistant to degradation [17].

We hypothesized that alveolar–capillary membrane distension and cyclic deformation is associated with the quantifiable presence of specific miRNAs. In addition, the analysis of the genes regulated by these miRNAs could help to identify additional mRNA signatures associated with injurious mechanical ventilation. To test these hypotheses, we analyzed several models of alveolar cell stretch to define miRNA and gene signatures related to lung overdistension. In addition, we validated the identified signatures in animal models of lung injury with abnormal stretch, in a clinically relevant ex vivo models using human lungs, in BALF from patients exposed to mechanical stretch and in serum samples of COVID-19 mechanically ventilated patients.

## Methods

A detailed description of the methods can be found in the online supplement.

### Study overview

**Fig. 1:**
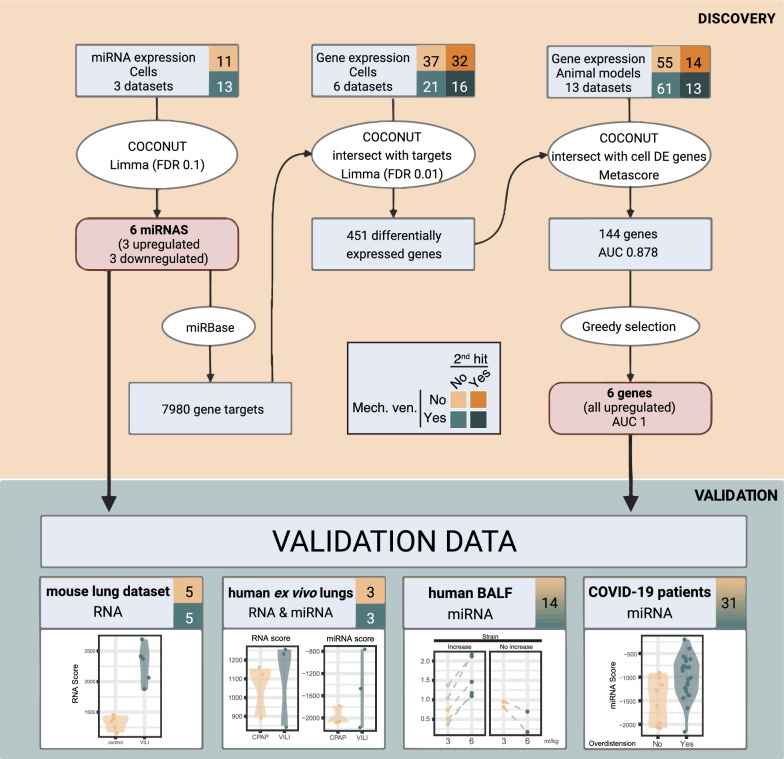
Study overview. Data sets containing miRNA expression after mechanical stretch in cell models were identified, pooled and analyzed. A signature including 6 miRNAs was identified. Genes regulated by these miRNAs were identified, and its expression studied in cell models, yielding a gene signature of 451 genes. Validation of this signature in animal models confirmed a differential expression of 144 genes. After application of a greedy algorithm, 6 genes predicted stretch with an area under the ROC curve of 1. Finally, both the miRNA and the gene signatures were tested in human lungs submitted to mechanical stretch and bronchoalveolar lavage fluid and serum from mechanically ventilated patients

The study overview is depicted in Fig. 1. We performed sequential pooled analyses aimed to identify microRNAs and genes involved in the response to cyclic stretch. In Step 1, data sets reporting changes in microRNA expression in cells exposed to static versus cyclic stretch conditions [18–20] were combined. This analysis yielded 6 microRNAs with differential expression after cyclic stretch. In step 2, data sets reporting changes in gene expression (mRNA) from lung epithelial cells exposed to cyclic stretch in-vitro [16, 21–25] were used to identify 451 putative microRNA targets that were differentially expressed by cyclic stretch. Step 3 of the discovery workflow was to determine expression of these 451 genes in animal models of VILI [26–37]. A transcriptomic score based on the expression of 144 genes was calculated, as well as its accuracy to identify mechanical stretch. This signature was optimized, obtaining a 6-gene, stretch-specific signature. Finally, we validated the miRNA and gene signatures in murine tissue, human lungs ventilated ex-vivo and in BALF and serum from mechanically ventilated patients (see online supplement for the experimental details of each study).

### Selection of studies

Data sets reporting microRNA or gene expression in models of cyclic stretch were identified in publicly available repositories (see Online Supplement for a detailed search strategy). Sixty-seven data sets were reviewed, and 22 studies included in the discovery phase (Tables 1, 2 and 3).

**Table 1:** Data sets measuring micro-RNA expression in epithelial cells submitted to cyclic stretch

Data set	Ref.	Platform	Cells	Intervention	*n*
GSE131645	[18]	GPL21527	Mouse MLE12	Static control 10% stretch, 24 h	4 2
GSE75100	[19]	GPL18058	Primary human alveolar epithelial type II	Static control 20% stretch, 48 h	3 3
GSE36256	[20]	GPL7722	Rat alveolar epithelial cells	Static control 25% stretch, 1 h 25% stretch, 6 h	4 4 4

**Table 2:** Data sets measuring gene expression in epithelial submitted to cyclic stretch, with or without a second mechanism of injury (second-hit)

Data set	Ref	Platform	Cells	Intervention	*n*
GSE59128	[21]	GPL10558	Primary human alveolar epithelial type II	Static control Mechanical injury, 0 h Mechanical injury, 8 h Mechanical injury, 24 h 20% stretch, 8 h 20% stretch, 24 h 20% stretch + mech. injury, 8 h 20% stretch + mech. injury, 24 h	24 8 8 4 4 4 4 4
GSE34789	[22]	GPL570	Human Calu-3	Static control 20% stretch, 18 h	1 1
GSE27128	[23]	GPL570	Human Calu-3	Static control 30% stretch, 24 h	3 3
GSE16650	[16]	GPL570	Human Beas-2b	Static control 5% Stretch 4 h LPS, 4 h TNF-α, 4 h 5% Stretch + LPS, 4 h 5% Stretch + TNF-α, 4 h	2 2 2 2 2 2
GSE1541	[24]	GPL201	Human A549	Static control 20% stretch, 1 h 20% stretch, 4 h LPS, 1 h LPS, 4 h TNF, 1 h TNF, 4 h 20% stretch + TNF-α, 1 h 20% stretch + TNF-α, 4 h	4 2 2 2 2 2 2 2 2
GSE3541	[25]	GPL341	Rat fetal alveolar epithelial type-II cells	Static control, 16 h 5% stretch, 16 h	3 3

*LPS* lipopolysaccharide, *TNF-α* tumor necrosis factor alpha

**Table 3:** Data sets measuring gene expression in animal models of lung injury involving mechanical stretch, with or without a second hit

Data set	Ref.	Platform	Animal	Intervention	*n*
GSE121550	[26]	GPL16570	Mouse	Spontaneous breathing MV, DP 15 cmH_2_O, 2.5 h	6 6
GSE85269	[27]	GPL16750	Mouse	Spontaneous breathing MV, (DP 15 cmH2O), 2.5 h	3 3
GSE18341	[28]	GPL1261	Mouse	Spontaneous breathing MV (VT 15 ml/Kg), 2 h Inhaled LPS MV (VT 15 ml/kg, 2 h) + Inhaled LPS	8 7 8 7
GSE11434	[29]	GPL1261	Mouse	Spontaneous breathing MV (DP 20 cmH2O), 3 h	5 5
GSE9368	[30]	GPL1261	Mouse	Spontaneous breathing MV (VT 30 ml/kg), 4 h	3 3
GSE9314	[30]	GPL1261	Mouse	Spontaneous breathing MV (VT 30 ml/kg), 4 h	4 4
GSE86229	[31]	GPL6246	Mouse	Spontaneous breathing MV (VT 35 ml/kg), 3 h	5 10
GSE31678	[32]	GPL1355	Rat	Spontaneous breathing MV (VT 18 ml/kg), 3 h	2 3
GSE7041	[33]	GPL1355	Rat	Spontaneous breathing MV (VT 20 ml/kg), 2 h	3 3
GSE9208	[34]	GPL8321	Mouse	Spontaneous breathing MV (VT 30 ml/kg), 2 h	3 3
GSE7742	[35]	GPL5145	Mouse	Spontaneous breathing MV (VT 10 ml/kg), 8 h	3 4
GSE2411	[36]	GPL339	Mouse	Spontaneous breathing MV (VT 10 ml/kg), 4 h Inhaled LPS MV (VT 10 ml/kg), 4 h + Inhaled LPS	6 6 6 6
GSE2368	[37]	GPL81 GPL85	Mouse Rat	Spontaneous breathing MV (VT 35 ml/kg), 2 h Spontaneous breathing MV (VT 12 ml/kg), 5 h	2 2 2 2

*MV* mechanical ventilation, *DP* driving pressure, *VT* tidal volume, *LPS* lipopolysaccharide

### Data set processing and differential expression analysis

Raw data was downloaded, processed and pooled using Combat CO-Normalization Using conTrols (COCONUT) algorithm [15]. The resulting pooled data sets can be explored using a web application available at https://crit-lab.shinyapps.io/stretch_signature_shinyapp/. Differences in microRNA or mRNA expression were calculated by fitting expression to a linear model including stretch and a second mechanism of injury (second-hit) as factors. Given the large number of features compared and, thus, the high risk of false positive results, *p* values were adjusted using the Benjamini–Hochberg procedure, assuming a false discovery rate of 5% [38]. MicroRNAs with a corrected *p* value lower than 0.1 (to avoid false negatives due to the small sample size) and mRNAs with a corrected *p* value lower than 0.01 were considered differentially expressed. Enriched pathways were identified by Gene Set Enrichment Analysis (GSEA) using the R package clusterprofiler [39].

### Transcriptomic scores

Transcriptomic signatures were defined as microRNA/gene sets with significant differences after stretch, and improved using a greedy algorithm. For a given signature, a transcriptomic score was computed for each sample as the geometric mean of the expression of upregulated genes minus the geometric mean of expression of the downregulated genes.

### Validation studies

Experimentally, the microRNA and gene signatures were validated in a murine model of ventilator-induced lung injury (ventilation with a tidal volume of 30 ml/Kg) and in an ex vivo human model (perfused lungs on CPAP (10cmH_2_O) or ventilated with 12 ml/kg predicted body weight (PBW) and zero PEEP [40]).

The microRNA signature was also validated in clinical samples. miRNAs were quantified in bronchoalveolar lavage from 7 patients (Supplementary Table 1) ventilated with a tidal volume of 6 and 3 ml/Kg PBW during venoarterial ECMO [41]. Overdistension was identified using lung strain (ratio between tidal and end-expiratory lung volume). In addition, a microRNA score was calculated in serum from mechanically ventilated COVID-19 patients (Supplementary Table 2) [42]. Arterial blood gas samples were recorded before and after a change in PEEP and overdistension defined as an increase in PaCO_2_ after an increase in PEEP or a decrease in PaCO_2_ after a decrease.

### Statistical analysis

Scores were compared among groups using a *T* test or an analysis of the variance. Post-hoc tests were done using Tukey’s HSD correction. Correlations were assessed using Spearman’s coefficient. Accuracy analyses were done by computing AUROC with its 95% confidence interval, which were compared against AUROCs obtained from scores calculated with random signatures by bootstrapping. AUROCs of the gene signature in animal studies were iteratively calculated excluding one study in each calculation (*leave-one-out*). All the analyses and plots were done using the statistical software R. All code is available at https://github.com/Crit-Lab/stretch_signature.

## Results

### Identification of a microRNA signature of cyclic stretch

**Fig. 2:**
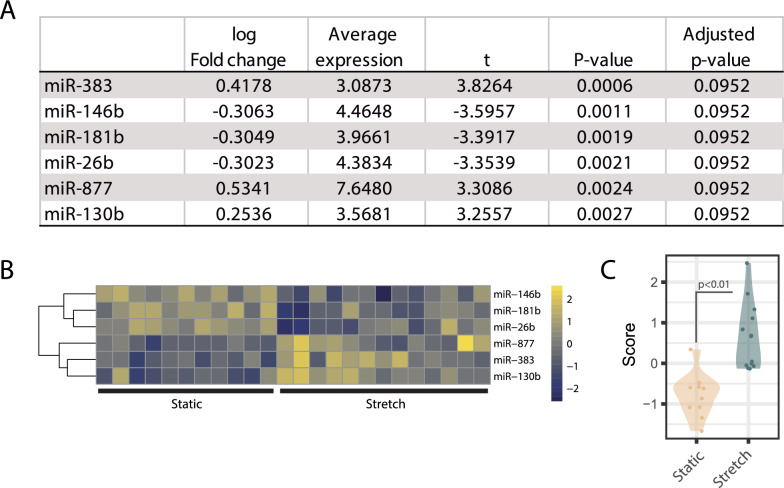
Identification of a micro-RNA signature of cyclic stretch. **A** MiRNAs with differential expression (adjusted *p* value < 0.1) between cells cultured in static conditions (*n* = 11) or after cyclic stretch (*n* = 13) were identified. **B** Heatmap of the identified miRNAs. **C** Transcriptomic score was calculated for each sample as the geometric mean of up-regulated miRNAs minus the geometric mean of the down-regulated miRNAs

Data from 3 data sets measuring microRNA expression in cells exposed to cyclic mechanical stretch (Table 1) were collected and normalized. Data sets were combined (*n* = 24 samples) and renormalized using the COCONUT algorithm (Supplementary Fig. 1). A total of 209 microRNAs were present in all three data sets. Among these, six were differentially expressed between stretched and non-stretched cells (upregulated: mir-383, mir-877, mir-130b; down-regulated: mir-146b, mir-181b, mir-26b, Fig. 2A, B). A transcriptomic score combining the expression of these microRNAs was higher in samples from stretched cells (Fig. 2C) and demonstrated excellent sensitivity and specificity to identify stretch, with an AUROC of 0.958 (0.872–1). Values for each microRNA and study are shown in Supplementary Fig. 2.

### Identification of a stretch microRNA-regulated gene signature

**Fig. 3:**
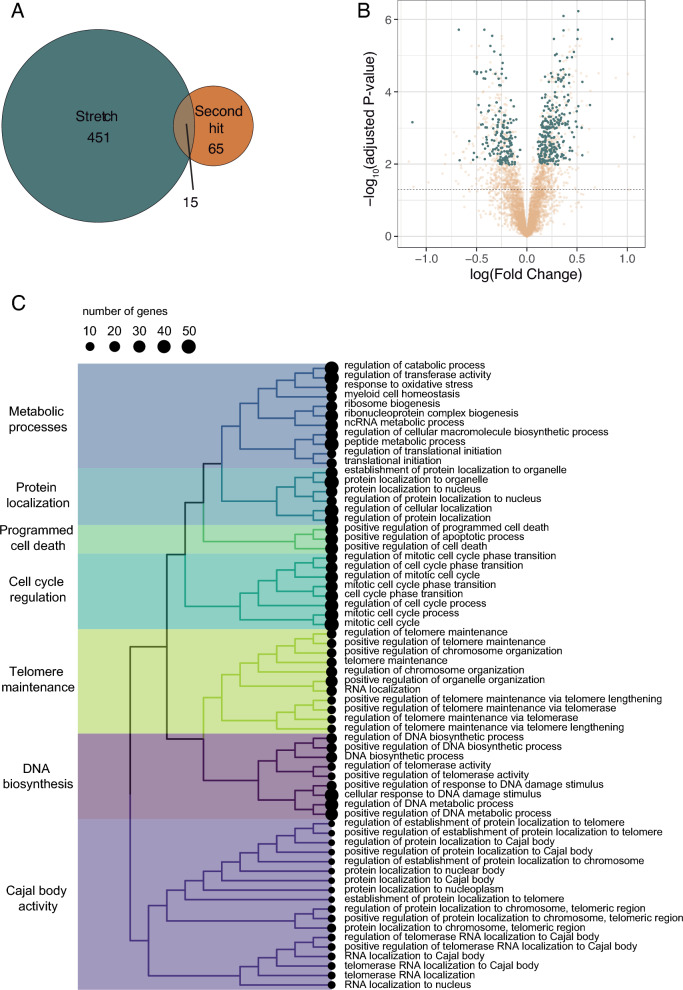
Identification of a gene signature dependent on stretch-regulated miRNAs. **A** Gene expression in cells cultured in static conditions (*n* = 37), after cyclic stretch (*n* = 21), a second-hit (*n* = 32) or stretch plus a second-hit (*n* = 16) was assessed. Among the genes regulated by stretch-dependent miRNAs, 41 were differentially expressed in cells only after mechanical stretch, 65 after other types of lung injury and 15 in both. **B** Volcano plot of gene expression after stretch. Those genes with significant differential expression and regulated by stretch-dependent miRNAs are highlighted in green. **C** Enriched pathways identified by a Gene Set Enrichment Analysis (GSEA). Each color in this tree-plot represents a major ontogeny group

Using available databases (miRecords, miRTarBase and TarBase), we identified 7980 experimentally validated gene targets of the 6 stretch-microRNAs. Expression of these target genes was assessed in 5 data sets (*n* = 106 samples) reporting data from cells exposed to cyclic mechanical stretch (Table 2). Some of these data sets also contained experimental groups with a second hit such as lipopolysaccharide (LPS). Data sets were pooled and normalized using the COCONUT algorithm (Supplementary Fig. 3). Differential gene expression was assessed using a linear model with two factors (cyclic stretch and the presence of a second hit). Among the 7980 genes regulated by the stretch-microRNAs, this model identified 451 genes differentially expressed between stretched and non-stretched cells, and 65 genes that were further differentially expressed by exposure to a second hit. Expression of 15 genes was modified by both factors (Fig. 3A). The position of stretch-responsive genes in the overall gene universe is shown in Fig. 3B. The enriched pathways corresponding to these genes according to the GSEA analysis include regulation of cell cycle and death and regulation of macromolecule trafficking (Fig. 3C).

### Validation and optimization of the ‘stretch-responsive’ gene signature in animal models

**Fig. 4:**
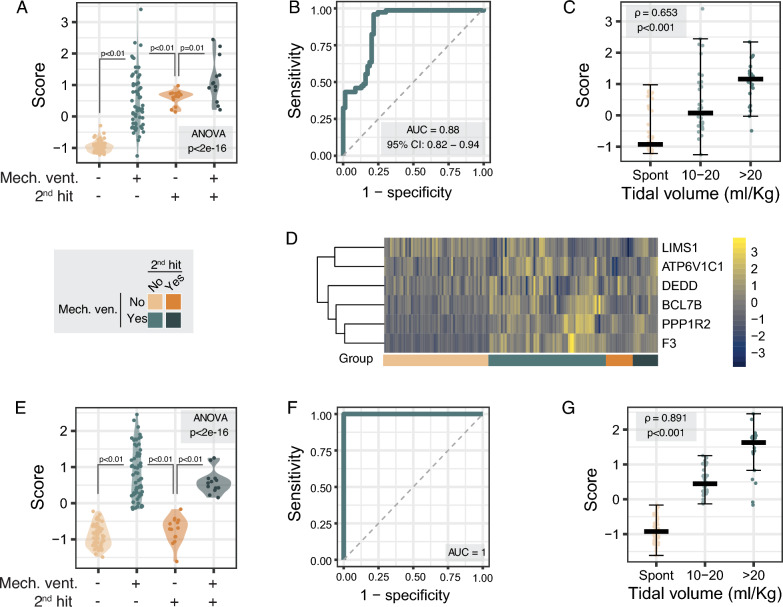
Validation of transcriptomic scores in animal models. **A** Transcriptomic scores of the identified genes were quantified in different models of lung injury including spontaneously breathing animals with intact lungs as controls (*n* = 55) and animals submitted to mechanical ventilation (*n* = 61), a second-hit (*n* = 14) or both (*n* = 13). **B** Accuracy of this score to identify mechanical stretch was studied using a ROC curve (**B**), with an area under the ROC curve of 0.88. **C** Transcriptomic score was proportional to the magnitude of the stretch tested (Spearman’s rho 0.653). **D** Heatmap of the six genes identified by a greedy algorithm and included in a specific signature. **E** Results of a transcriptomic score calculated using only these six genes. **F** Accuracy of the transcriptomic score to identify stretch (AUROC = 1). **G** Correlation between the transcriptomic score and tidal volume (Spearman’s rho 0.891)

To validate this gene signature, data from 14 different transcriptomic data sets looking at changes in gene expression in animal models of lung injury involving injurious mechanical ventilation (Table 3) were downloaded from GEO, combined (*n* = 143 samples) and normalized with COCONUT (Supplementary Fig. 4). Data for a total of 144 genes from the original signature were available from all data sets, and their expression is shown in Supplementary Fig. 5. A transcriptomic score was calculated for each sample. Groups of animals exposed to mechanical ventilation showed higher scores than those breathing spontaneously (Fig. 4A). This transcriptomic score had an AUROC of 0.878 (95% CI 0.819–0.938) to detect mechanical stretch in the entire data set (Fig. 4B). The probability of an AUROC higher than 0.9 using any random 144-gene signature was < 0.001 (Supplementary Fig. 6). The score correlated with the magnitude of stretch (Spearman’s *ρ* = 0.653, *p* < 0.001 for the linear correlation, Fig. 4C).

The accuracy of this gene signature was improved using a selection of the most informative features using a greedy algorithm aimed to achieve the best AUROC. This selection yields a signature of 6 upregulated genes (*Lims1*, *Atp6v1c1*, *Dedd*, *Bclb7*, *Ppp1r2* and *F3,* Fig. 4D). Expression of these genes in each condition in cell and animal studies is shown in Supplementary Figs. 7 and 8, respectively. A transcriptomic score calculated using only these genes showed significant differences between ventilated and non-ventilated animals (Fig. 4E) and an excellent accuracy for stretch detection (Fig. 4F), with an AUROC of 1 (Fig. 4F). AUROCs for each gene are provided in Supplementary Fig. 9. Supplementary Fig. 10 shows the values of the calculated scores for each study. Iterative analyses of these AUROCs excluding one study in each calculation (leave-one-out) yielded similar values, within the confidence interval of the original AUROC (Supplementary Fig. 11). Using random 6-gene signatures, the probability of an AUROC above 0.97 was < 0.001 (Supplementary Fig. 12). Again, scores were linearly correlated with the magnitude of stretch (Spearman’s *ρ* = 0.891, *p* < 0.001 for the linear correlation, Fig. 4G).

### External validation of the signature in animal and human samples

**Fig. 5:**
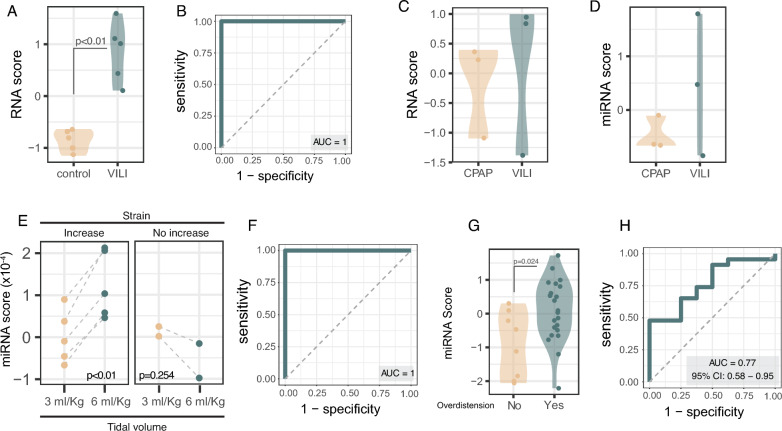
Validation of transcriptomic signatures. **A**, **B** Transcriptomic scores (**A**) computed using the 6-gene signature in mice (5 on spontaneous breathing and 5 ventilated with a tidal volume of 30 ml/kg), with the corresponding ROC curve (**B**). **C**, **D** Gene (**C**) and miRNA (**D**) scores in six human lungs ventilated only with CPAP (*n* = 3) or with a tidal volume of 12 ml/kg (ventilator-induced lung injury, VILI, *n* = 3). **E**–**H** miRNA signature was also validated in two clinical settings. First, in BALF from 7 patients ventilated with tidal volumes of 3 and 6 ml/Kg and supported with venoarterial ECMO, and with lung strain measured as a marker of overdistension (**E**). The accuracy of this miRNA score to detect an increase in strain was 1 (**F**). The same miRNA signature was measured serum from mechanically ventilated COVID-19 patients, and scores (**G**) and AUROC (**H**) calculated

Several validation analyses were performed to test the validity of the identified signatures. First, experimental models of ventilator-induced lung injury in animals and human lungs were analyzed. In these settings, the 6-gene transcriptomic score was higher in animals ventilated with high tidal volume compared to those spontaneously breathing (*n* = 5 per group, Fig. 5A), with an AUROC of 1 (Fig. 5B). The signatures were also measured in ex-vivo ventilated human lungs (*n* = 3 per group). Both the gene (Fig. 5C) and microRNA (Fig. 5D) scores were higher in two out of the three samples with VILI. Due to the reduced sample size, no statistical analyses were performed. Individual values of miRNAs and genes for these samples are shown in Supplementary Fig. 13.

Then, the scores were tested in clinical scenarios. BALF from 7 mechanically ventilated patients with respiratory failure and supported with venoarterial ECMO was obtained after ventilation with tidal volumes of 3 and 6 ml/kg. Increasing tidal volume from 3 to 6 ml/kg increased lung strain, a marker of lung overstretching, in 5 out 7 patients. MicroRNAs were quantified in BALF samples (Supplementary Fig. 14) and the corresponding miRNA score calculated. The obtained scores increased only in those patients in which strain increased during ventilation with 6 ml/Kg (Fig. 5E). These results yield an AUROC of 1 (Fig. 5F).

Finally, the accuracy of the miRNA signature quantified in serum samples was tested in a cohort of 31 COVID-19 mechanically ventilated patients. Overdistension was defined using changes in PaCO_2_ after modification of PEEP. Patients with overdistension (*n* = 23) showed higher scores than those without (Fig. 5G). In this setting, the AUROC of the signature to identify stretch is 0.766 (0.582–0.950) (Fig. 5H).

## Discussion

Our results show that two transcriptomic signatures comprising 6 miRNAs and 6 genes, respectively, are capable to specifically identify lung stretch. Transcriptomic scores computed using these signatures are proportional to the amount of stretch and showed good accuracy in animal models, a clinically relevant ex vivo model using human lungs and in BALF or serum from ventilated patients. These results open the possibility of biological monitoring of lung overdistension.

Injured lungs are at risk of further deterioration due to overstretching during mechanical ventilation [43]. Consequently, avoidance of VILI is a major goal of respiratory support in this population. A tidal volume of 6 ml per kg of predicted body weight (adjusted to height and sex) is the basis of protective ventilatory strategies. Further refinements, including limitation of driving pressure to 15 cmH_2_O or plateau pressure to less than 30 cmH_2_O [44, 45], have been proposed but not tested in clinical trials. However, none of these considers the amount of lung available for ventilation or the occurrence of local hyperinflation. The inability to detect patients at highest risk of lung overdistension may, in part, explain the lack of a beneficial effect with the use of lower tidal volume ventilation facilitated by extracorporeal carbon dioxide removal in a broad population of patients with acute hypoxemic respiratory failure [41, 46]. Measurements of functional residual capacity and adjustment of tidal volume to minimize strain (the ratio between tidal volume and functional residual volume) have been proposed [47], but sound clinical evidence is lacking. Moreover, fine tuning of mechanical ventilation is aimed to avoid not only gross VILI (in terms of barotrauma), but more subtle consequences, such as triggering local inflammation or the systemic spread of the response. In this sense, biomarkers could complement the previously described measurements and help the clinicians to assess the host response to ventilation.

Our work provides a framework for such biological monitoring. Other transcriptomic signatures have successfully contributed to the diagnosis of specific forms of lung injury, such as viral infections [48]. Inclusion of experimental models with more than 1 hit allowed us to isolate miRNAs and genes specific for stretch, discarding the changes in expression related to unspecific damage. Moreover, the calculated score shows a linear correlation with the magnitude of stretch, reinforcing its value as biomarker of the transmitted mechanical load, and discarding other phenomena such as recruitment that would result in a decrease in the magnitude of cell deformation (as the same tidal volume is distributed in an increased static lung volume). By these reasons, the identified scores could be used even in cases of heterogeneous injury (in a similar fashion to troponins to measure cardiac damage) and in global samples, such as BALF, serum or exhaled breath condensate, although standardized safety thresholds are to be defined.

Several of miRNAs identified have been described in models of lung injury or may modulate the pathogenesis of VILI. miR-383 regulates cell apoptosis in alveolar cells [49]. miR-130b has an anti-inflammatory role in acute lung injury by decreasing TGFβ signaling [50] and repressing macrophage M1 polarization [51]. miR-877 is a negative regulator of alveolar cell proliferation [52], in line with our pathway analysis, and inhibits bleomycin-induced lung fibrosis [53]. Interestingly, miR-146b [54, 55], miR-181b [56] and miR-26b [57, 58] have anti-inflammatory effects in different models of lung injury, so their decrease could explain the pro-inflammatory response related to lung stretch.

Regarding the 6-gene signature, only F3 has been linked to acute lung injury and mechanical stretch. It has been reported that tissue factor (the product of the gene F3) increases in response to alveolar stretch [59, 60] and in mechanically ventilated animals [61]. This molecule may play a relevant role in lung injury [62]. None of the remaining genes have been described in models of ventilator-induced lung injury, but they are known regulators of the underlying cell responses to stretch. Several of these genes act as regulators of apoptosis, such as *DEDD* [63] and *BCLB7* [64], and cell cycle progression (including *DEDD* [65] and *PPP1R2* [66]). Other genes have been involved in cytoskeleton arrangement and cell–matrix interactions. *LIMS1* encodes PINCH-1, a regulator of cell adhesion mediated by integrins [67], and *ATP6V1C1* encodes a lysosomal proton pump that, by modifying the intracellular environment, may regulate autophagy [68] and cytoskeleton arrangement [69]. However, the defining feature of the identified miRNAs and genes could be their mechanosensitivity (i.e., their expression in response to mechanical stretch) rather than a pathogenetic role in this setting.

Our work has some limitations. The signature has been obtained by combining different studies, with different methodologies and even in different species. Although the pathogenetic mechanisms triggered by lung injury and overdistension may be different across species, the use of heterogeneous data has been proposed as a strategy to identify highly conserved, specific signatures [70]. Indeed, the number of experimental models tested is limited, and we cannot discard that other causes of lung injury may recapitulate the described signatures, thus decreasing their specificity. We tried to overcome some of these limitations by combining the power of stretch regulation at two molecular levels, miRNA and mRNA, to maximize the chances of detecting true biology. In addition, we only considered epithelial cells, due to the lack of consistent data sets from other lung cell lineages, such as fibroblasts or endothelial cells.

The validation phase also has its limitations. The used data sets do not allow to differentiate among lung zones, in which the distribution of forces may be heterogeneous, and there is no distinction between controlled and assisted ventilation, so the role of inspiratory efforts cannot be addressed. There is evidence that this anisotropic distribution of stretch occurs even at the alveolar level [8]. Therefore, the measurements must be considered an average, and the significance at regional scales must be explored. Finally, our validation in human samples is limited by a small sample size. It must be noted that the ex vivo model is valuable as it provides strong evidence of the effects of VILI in human lungs. The analysis in BALF and serum samples, although more representative of the clinical setting, is, however, limited by the inherent difficulties of BALF monitoring in clinical practice and, more importantly, the lack of a clear gold standard for lung overstretching. We used strain as a marker of injurious settings, as this magnitude is related to lung inflammation [47], although is rarely available at the bedside. Changes in PaCO_2_ in response to PEEP, although easy to perform, may have less accuracy. Of note, most of the patients in which overdistention was detected using the signature and confirmed by changes in strain or PaCO_2_ had plateau pressures below the recommended threshold of 28–30 cmH_2_O. A large-scale clinical validation using conventional samples (i.e., blood or BALF) and relevant endpoints (such as development of new or additional lung damage) is needed to define the usefulness of the signatures in patients.

## Conclusions

We have identified two transcriptomic signatures of miRNAs and genes that could correlate with lung overstretch. Although their validity must be confirmed in clinical settings, they constitute a potential approach to monitor the biological response to stretch and open the possibility for a biomarker-based setting of mechanical ventilation.

## Supplementary Information

Supplementary Material 1.

## Data Availability

Data sets used in the study are publicly available at Gene Expression Omnibus (https://www.ncbi.nlm.nih.gov/geo/), using the references provided in Tables 1, 2 and 3. The resulting pooled data sets can be explored using a web application available at https://crit-lab.shinyapps.io/stretch_signature_shinyapp/. All code and data used for analysis is available at https://github.com/Crit-Lab/stretch_signature.

## Abbreviations

AUROC: Area under the receiver–operator curve
BALF: Bronchoalveolar lavage fluid
COCONUT: COMBAT Co-normalization using controls
CPAP: Continuous positive airway pressure
ECMO: Extracorporeal membrane oxygenation
FDR: False discovery rate
GSEA: Gene set enrichment analysis
LPS: Lipopolysaccharide
PBW: Predicted body weight
PEEP: Positive end-expiratory pressure
RMA: Robust multi-array average
UMIs: Unique molecular identifiers
VILI: Ventilator-induced lung injury
