# Supplementary material for: Prediction of lung overdistension during mechanical ventilation using micro-RNA and gene expression
Source: Intensive Care Med Exp. 2025 Jun 7;13:60. doi: 10.1186/s40635-025-00768-2 (PMC12145361; doi:10.1186/s40635-025-00768-2)
Supplement: Supplementary file 1 — Supplementary Material 1. [file 40635_2025_768_MOESM1_ESM.pdf]

Prediction of lung overdistension during mechanical ventilation using micro RNA and gene expression

Cecilia López-Martínez, Paula Martín-Vicente, Laura Amado-Rodríguez, Inés López-Alonso, Margarita Fernández, Adrián González-López, Pablo Martínez-Camblor, Juan Gómez, Andrew J Boyle, Cecilia M O'Kane, Daniel F McAuley, James N Tsoporis, Claudia dos Santos, Guillermo M Albaiceta.

Online data supplement

Methods

Selection of studies

Datasets reporting microRNA or gene expression in models of cyclic stretch were identified in publicly available repositories (GEO: https://www.ncbi.nlm.nih.gov/geo/; ArrayExpress: https://www.ebi.ac.uk/arrayexpress/). The following search terms were used: (("stretch" OR "cyclic strain") AND ("Lung" OR "alveolar")) OR "Mechanical ventilation" OR "Ventilator induced lung injury". After the search, 67 datasets were manually reviewed. Studies lacking a control group with either intact cells cultured in static conditions or spontaneously breathing animals with normal lungs were excluded. A total of 22 studies were included in Step 1, Step 2 and Step 3 of the discovery phase of the study (Tables 1, 2 and 3 in the main text).

All the ventilated animals were deeply sedated and, in some cases, paralyzed. Spontaneously breathing animals were not sedated and did not receive any kind of assisted ventilation.

Dataset processing

Raw data was downloaded and processed (background correcting and normalization) using the Robust Multiarray Average (RMA) method (for Affymetrix arrays) or normal-exponential background correction followed by quantile normalization (all the other platforms). If raw data was not available (datasets GSE7742 and GSE2411), processed data was downloaded from the GEO website.

Datasets were pooled using Combat CO-Normalization Using conTrols (COCONUT) [1]. This method uses the ComBat algorithm (normalization of each gene expression using an empirical Bayes method) only in control samples, and applies the obtained normalization

parameters to the diseased samples. To fulfill the requirement that all the control samples represent the same distribution, only intact cells cultured in static conditions or animals with normal lungs and spontaneous breathing were considered as controls.

Differential miRNA and mRNA expression analysis

Differences in microRNA or mRNA expression were calculated by fitting COCONUT-normalized feature expression to a linear model including stretch and a second mechanism of injury (second-hit) as factors, and computing the F statistics with Bayesian moderation of the standard errors. P values were adjusted using the Benjamini-Hochberg correction (False Discovery Rate, FDR). MicroRNAs with a corrected p-value lower than 0.1 (to avoid false negatives due to the small sample size) and mRNAs with a corrected p-value lower than 0.01 were considered differentially expressed. Enriched pathways including these differentially expressed genes were identified by Gene Set Enrichment Analysis (GSEA) using the R package clusterprofiler [2].

Identification of microRNA target genes

Genes targeted by specific microRNAs were obtained after a systematic search in three available databases: miRecords (http://miRecords.umn.edu/miRecords)[3], miRTarBase (https://mirtarbase.cuhk.edu.cn/) [4] and TarBase (https://dianalab.e-ce.uth.gr/tarbasev8/index) [5] using the *multiMiR* package for R [6]. Only genes with experimental validation of targeting were considered.

Transcriptomic scores

For a given microRNA/gene signature, a transcriptomic score was computed for each sample as the geometric mean of the expression of upregulated genes expression minus the geometric mean of expression of the downregulated genes.

Greedy forward gene selection

To select the optimal set of features that identify mechanical stretch, a greedy forward algorithm aimed to improve the area under the receiver-operator curve (AUROC) was applied to the original set of differentially expressed features. The algorithm starts with the most significant feature (gene), and iteratively adds features, selecting the combination that results in the highest AUROC. This allows the identification of the subset of features that best differentiates between samples exposed to mechanical ventilation and those in spontaneous breathing.

Validation in an animal model

The gene signature was validated in an animal model of ventilator-induced lung injury in mice, for which RNAseq data is reported (GSE114132, available at https://www.ncbi.nlm.nih.gov/geo/query/acc.cgi?acc=GSE114132). Raw counts were downloaded, normalized using DEseq2 and transcriptomic scores and their AUROC were calculated.

Ex vivo human lung model

Lungs not suitable for transplantation, but consented for use in research, were obtained from UK organ donors. The project was approved by the London Central Research Ethics Committee (project reference 14/LO/0250). After retrieval, lungs were transported to the

laboratory, re-warmed with a perfusate solution of 5% albumin in DMEM before inflation, using a Vivoline LS1 *ex vivo* lung perfusion machine, with the oxygenator and leucocyte filter removed [7]. Whole blood was added to the perfusate solution to a final concentration of 10% and the lungs then either maintained on CPAP (10cmH_2O) with FiO_2 of 0.95 and a $FiCO_2$ of 0.05 or ventilated with 12ml/kg PBW and zero PEEP at a rate of 15 breaths per minute, and FiO_2 0.95/$FiCO_2$ 0.05 for 4 hours. These ventilatory settings were selected to either prevent or cause alveolar overdistension based on previous experiments [8]. After 4 hours the lungs were deflated, removed from the ventilation and perfusion circuit and a portion of tissue homogenized in TRI Reagent (Thermo Fisher, USA). The homogenate was incubated with isopropanol, washed with ethanol and the extracted RNA resuspended in RNase-free water. For microRNA extraction, total RNA was separated in a 15% urea-polyacrylamide gel, and 18-30nt RNA were cut out and eluted. Extracted microRNAs were sequenced using unique molecular identifiers (UMIs) to avoid undesirable PCR duplicates. RNA and Small RNAs were sequenced and processed for quality check and removal of adapters (BGI, China). Clean reads are available at GEO (https://www.ncbi.nlm.nih.gov/geo, accession number GSE173803).

Count matrices for the mRNA sequencing were obtained using the Salmon pseudoaligner [9] with the GRCh38 genome as reference. Samples were normalized using DESeq2 [10] and the RNA transcriptomic score was calculated for each sample. miRNA sequencing results were quantified using miRDeep2. A reference transcriptome containing miRNA sequences was obtained from http://www.mirbase.org/ftp.shtml)[11]. Normalized counts were used to calculate the miRNA score for each sample.

Bronchoalveolar lavage fluid samples

The protocol was reviewed and approved by the Regional Ethics Committee (Comité de ética de la investigación Clinica del Principado de Asturias, Spain, reference 22/17). After signed informed consent from patients' next of kin, a bronchoalveolar lavage was performed in 7 patients included in a clinical trial testing tidal volume of 6 and 3 ml/Kg predicted body weight (PBW) during venoarterial ECMO for cardiogenic shock [12]. Demographical and clinical data for this cohort is provided in Supplementary Table 1. Briefly, after 24 hours ventilated with these tidal volumes, end-expiratory lung volume was measured by nitrogen wash-in/wash-out (CareStation, General Electric, USA), and BALF recovered after instillation of 10 ml of sterile saline. Lung strain was calculated for each tidal volume as the ratio between tidal and end-expiratory lung volumes. The recovered BALF was filtered and stored at -80°C. MicroRNAs were measured in these samples (EdgeSeq miRNA whole transcriptomic assay, HTG Molecular Diagnostics Inc, USA).

Mechanically ventilated patients

To validate the feasibility of stretch quantification using peripheral blood, we analyzed a cohort of mechanically ventilated COVID-19 patients in which serum miRNA abundance were quantified. Data on this cohort has been published and is publicly available [13], and demographical and clinical data for this cohort is provided in Supplementary Table 2. . The miRNA signature was calculated and arterial blood gas samples were recorded before and after the closest change in PEEP. We defined overdistension as an increase in $PaCO_2$ after an increase in PEEP or a decrease in $PaCO_2$ after a decrease. In the short term, other mehcanisms that may modify $PaCO_2$, such as abrupt changes in blood flow, can be excluded, so the change in $PaCO_2$ reflect a decrease in ventilated alveolar volume,

probably due to an increase in alveolar dead space caused by overdistension. miRNA scores were compared between patients with and without overdistension.

Statistical analysis

Transcriptomic scores were compared among groups using a T-test or an analysis of the variance (in case of more than two groups). Normal distribution was assessed using a Shapiro-Wilk test. When appropriate, post-hoc tests were done using Tukey's HSD correction. Paired samples were compared using a T test for paired data. Correlations were assessed using Spearman's coefficient. Accuracy analyses were done by computing AUROC. The 95% confidence interval for AUROC was computed using the DeLong method. Obtained AUROCs were compared to a distribution of 10000 AUROCs obtained from scores calculated with random signatures of the same number of genes as the reference signature. As a sensitivity analyses, AUROCs of the gene signature in animal studies were iteratively calculated excluding one study in each calculation (*leave-one-out*). All the analyses and plots were done using the statistical software R (R Core Team, Vienna, Austria), including the packages *oligo*[14], *limma*[15], *MetaIntegrator*[16], *COCONUT*[1], *FSelectorRcpp*, *tidyr*, *clusterprofiler* [2], *ggplot2*[17] and *pROC*[18].

All code is available at https://github.com/Crit-Lab/stretch_signature.

Supplementary Table 1. Demographical and clinical data of patients with BALF sampling during conventional ventilation with a tidal volume of 6 ml/Kg of predicted body weight and under extracorporeal support. Values are reported as median (interquartile range) or as frequency.

Age (years)	61 [54 – 68]
Sex	3 female / 4 male
SAPS-3	67 [57 – 75]
PaO_2 / FiO_2	251 [179 – 278]
$PaCO_2$ (mmHg)	35 [32 – 37]
pH	7.43 [7.42 – 7.46]
Tidal volume (ml)	390 [370 – 450]
Positive end-expiratory pressure (cmH_2O)	7 [6 – 8]
Plateau pressure (cmH_2O)	18 [17 – 19]

Supplementary Table 2. Demographical and clinical data of COVID-19 patients. Values are reported as median (interquartile range) or as frequency.

	With overdistension	Without overdistension	
Age	61 (60 - 71)	70 (68 - 77)	0.172
Sex			
Male	18	8	0.85
Female	5	1	
Race			
White	22	7	0.195
Latin	0	1	
Black	1	0	
Hyypertension	16	5	0.737
Diabetes	3	1	1
COPD	3	1	1
BMI (Kg/m^2)	27.2 (26.6 - 32.47)	29.1 (25.69 - 32.2)	0.902
FiO_2	0.4 (0.4 - 0.6)	0.5 (0.4 - 0.6)	0.865
PaO_2/FiO_2	189.4 (183 - 235)	218 (152 - 266)	0.753
pCO_2 (mmHg)	44 (42 - 51)	43 (41 - 46)	0.276
pH	7.32 (7.31 - 7.34)	7.36 (7.33 - 7.39)	0.089
Respiratory rate (min^{-1})	18 (14 - 22)	18 (16 - 20)	0.815
Tidal volumen (ml)	488 (474 - 500)	491 (444.5 - 507)	0.516
Tidal volume/PBW (ml/Kg)	6.5 (5.6 - 6.7)	6.1 (5.3 - 6.7)	0.530
Plateau pressure (cmH_2O)	29 (27 - 32)	25 (22 - 28)	0.020
PEEP (cmH_2O)	14 (12 - 16)	12 (10 - 14)	0.093
Driving pressure (cmH_2O)	14 (13 - 18)	13 (10 - 15)	0.179
Respiratory system compliance (ml/cmH_2O)	33 (28 - 36)	38 (30 - 45)	0.256

Supplementary figures

Figure S1. Density plots of micro-RNA expression for each sample, before and after normalization using the COCONUT algorithm. Controls included exclusively cells cultured in static conditions, without any other further injury. Cases included cells submitted to cyclic stretch.

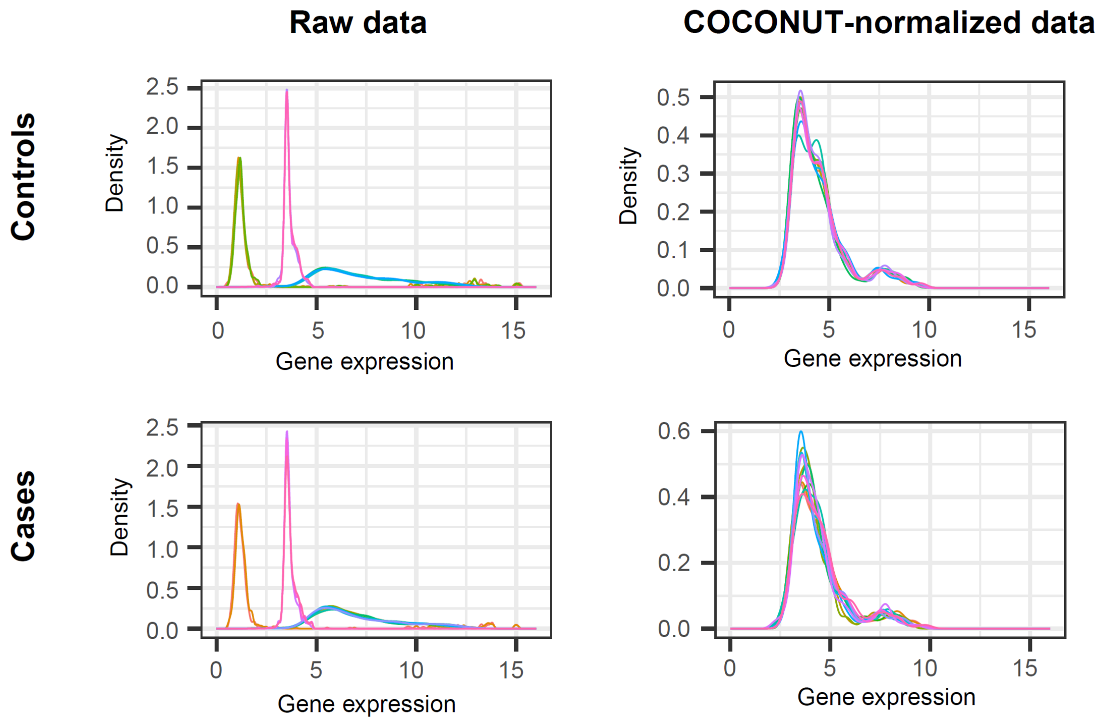

Figure S2. Abundance of microRNAs of interest in control and stretched cells in each study.

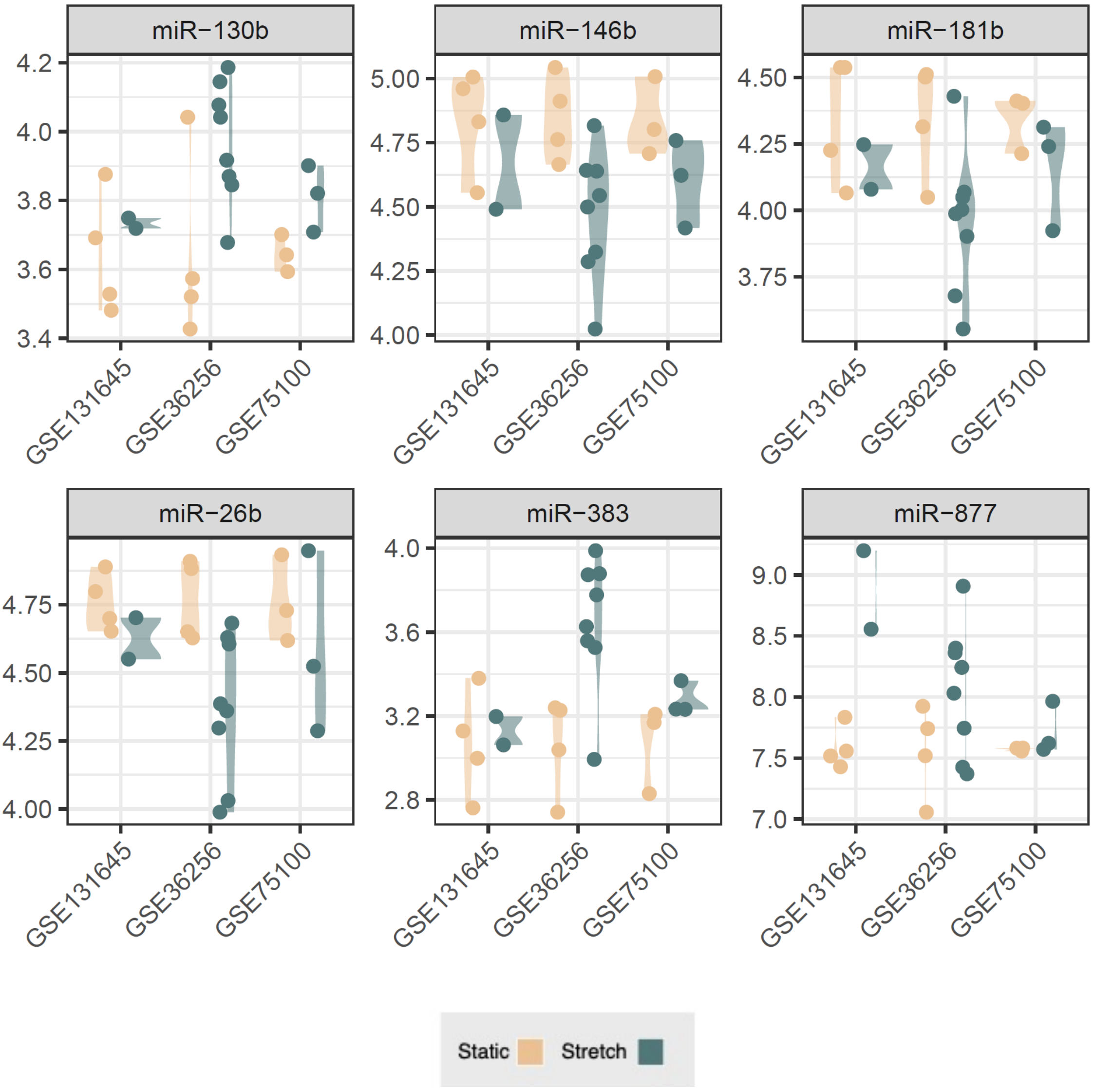

Figure S3. Density plots of gene expression for each sample, before and after normalization using the COCONUT algorithm. Controls included exclusively cells cultured in static conditions, without any other further injury. Cases included cells submitted to cyclic stretch, a second mechanism of injury (second-hit) or both.

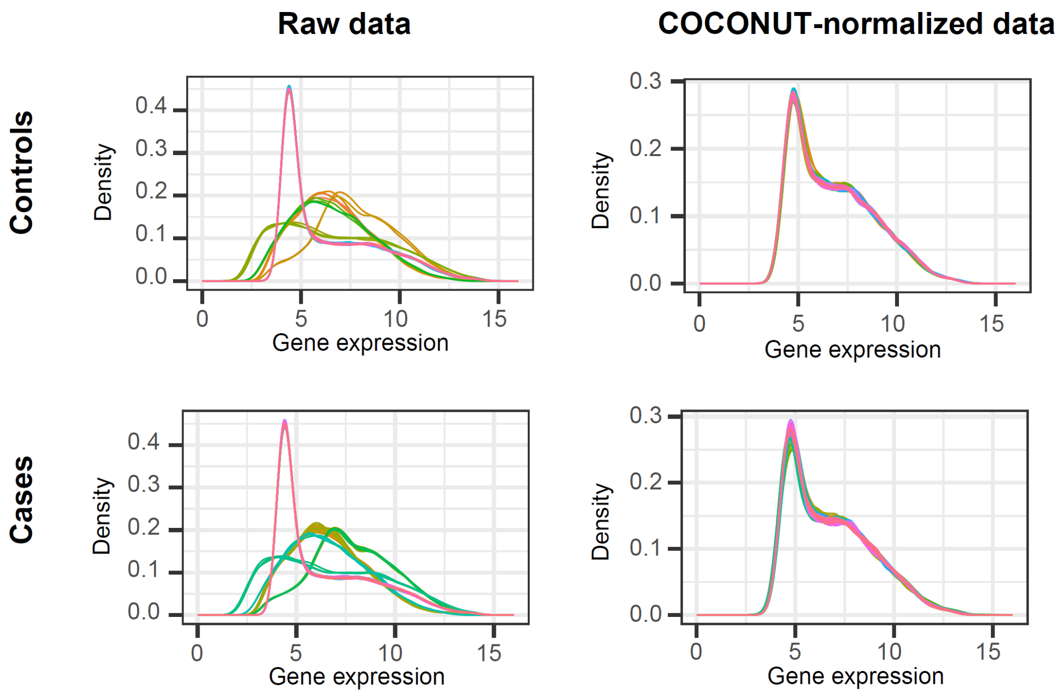

Figure S4. Density plots of gene expression for each sample, before and after normalization using the COCONUT algorithm. Controls included exclusively spontaneously breathing animals, without any other further injury. Cases included animals submitted to mechanical ventilation, a second mechanism of injury (second-hit) or both.

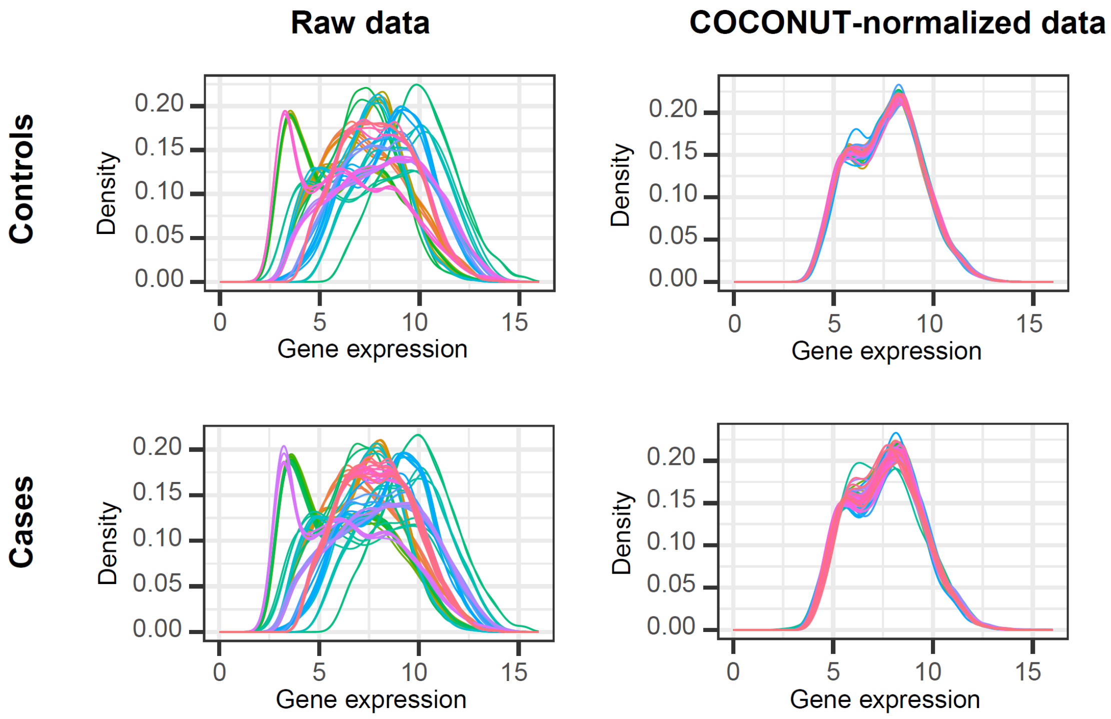

Figure S5. Heatmap showing expression of the 144 stretch-sensitive genes identified in cell stretch experiments in lung tissue from animal models of injury, including spontaneously breathing controls and animals submitted to mechanical ventilation, a second mechanism of injury (second-hit) or both.

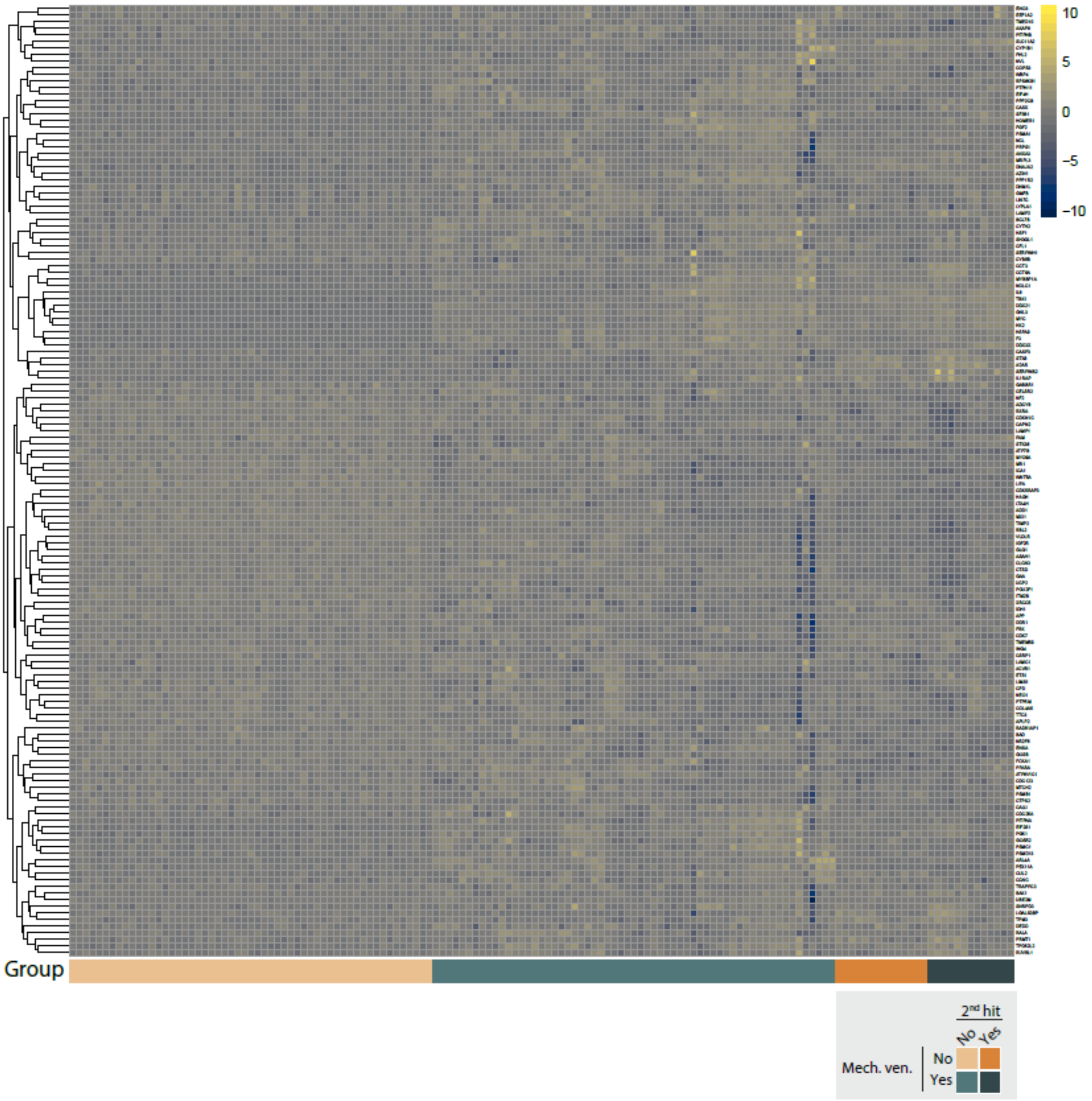

Figure S6. Distribution of area under the ROC curve (AUROC) of 10000 metascores generated using sets of 144 randomly chosen genes, to identify mechanically ventilated samples.

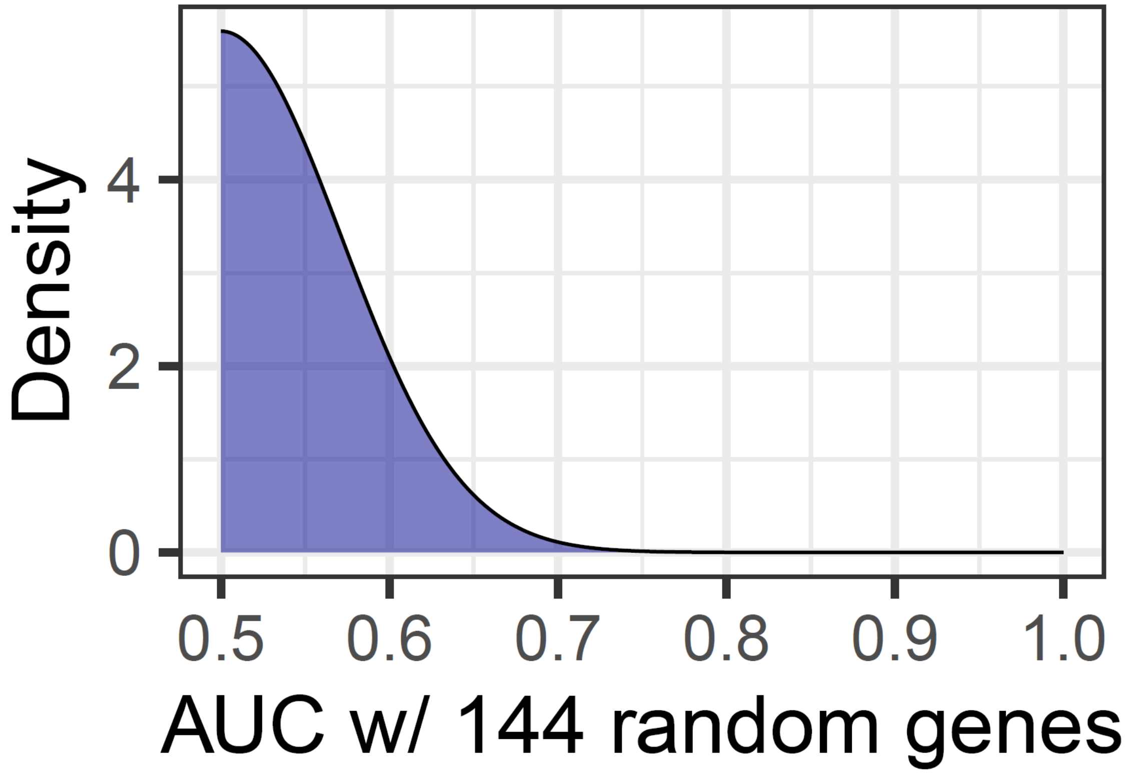

Figure S7. Expression of genes included in the stretch signature in studies of cell stretch.

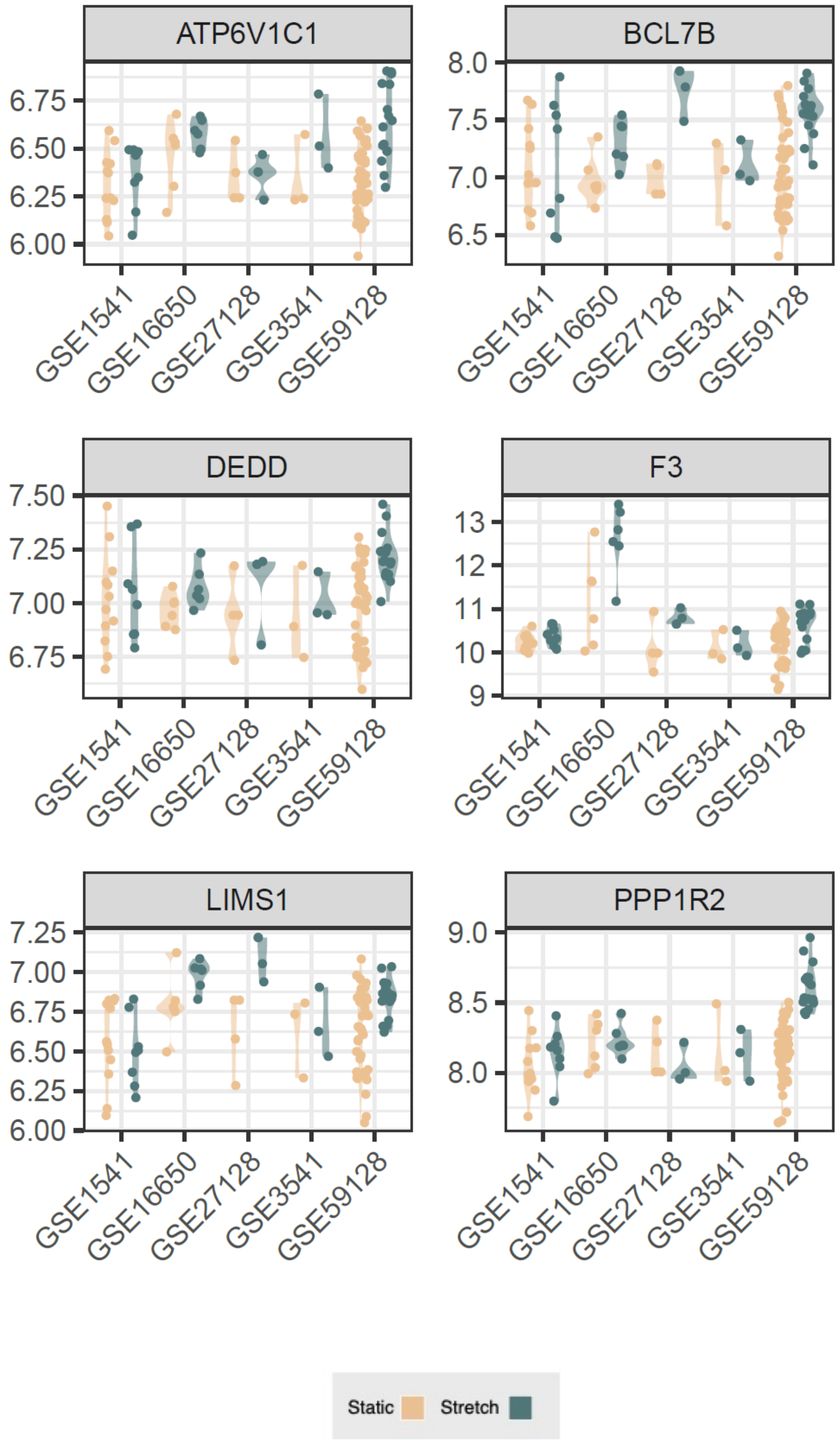

Figure S8. Expression of genes included in the stretch signature in animal models of lung overdistension.

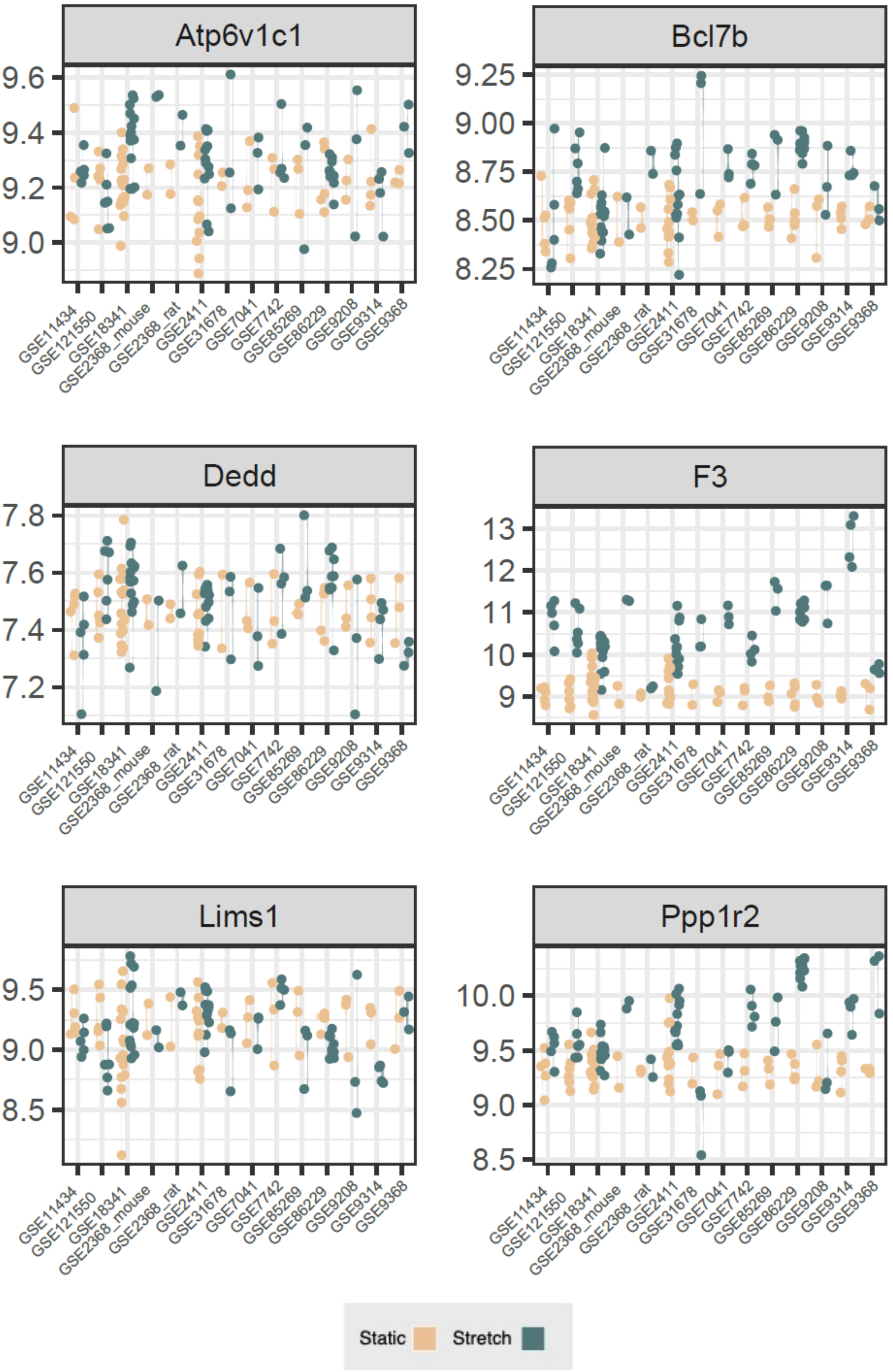

Figure S9. Individual AUROCs to detect lung streth in animal models for each gene included in the transcriptomic signature.

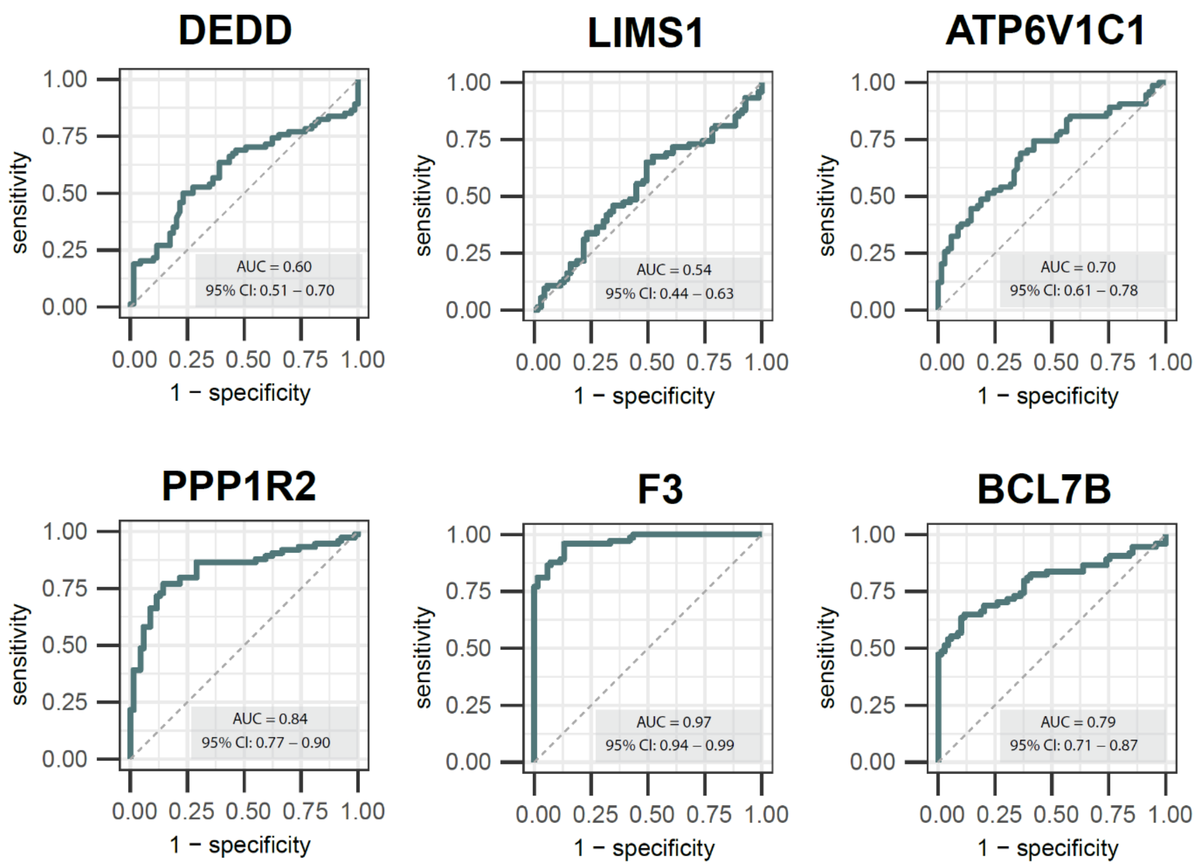

Figure S10. Transcriptomic scores calculated using a 4-gene signature in each animal study using an *in-vivo* lung stretch model.

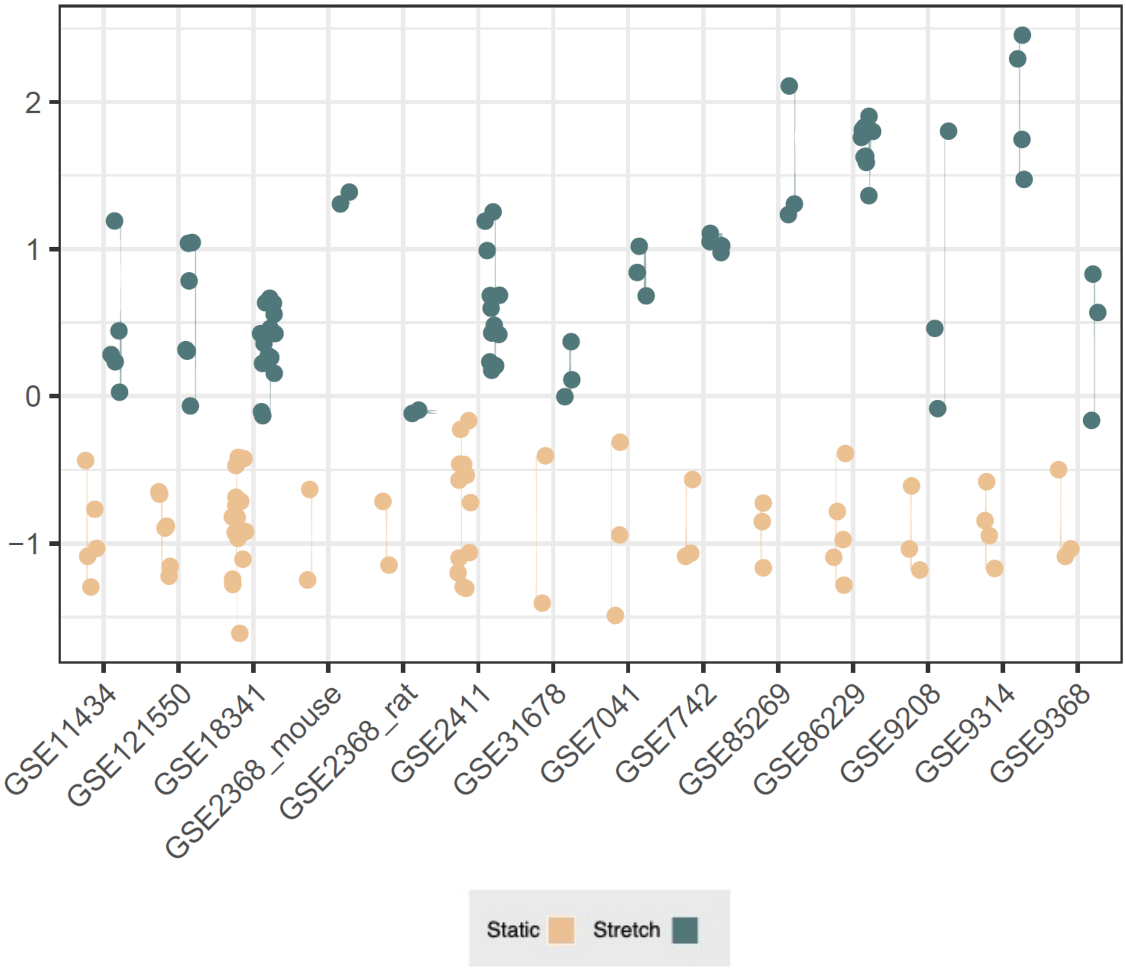

Figure S11. Leave-one-out analysis. Calculation of the area under the ROC curve of a 4-gene signature was iteratively repeated excluding one study in each calculation. Dots and bars represent the AUROC and the 95% confidence interval. The gray rectangle represents the 95% confidence interval of the AUROC calculated using all the studies.

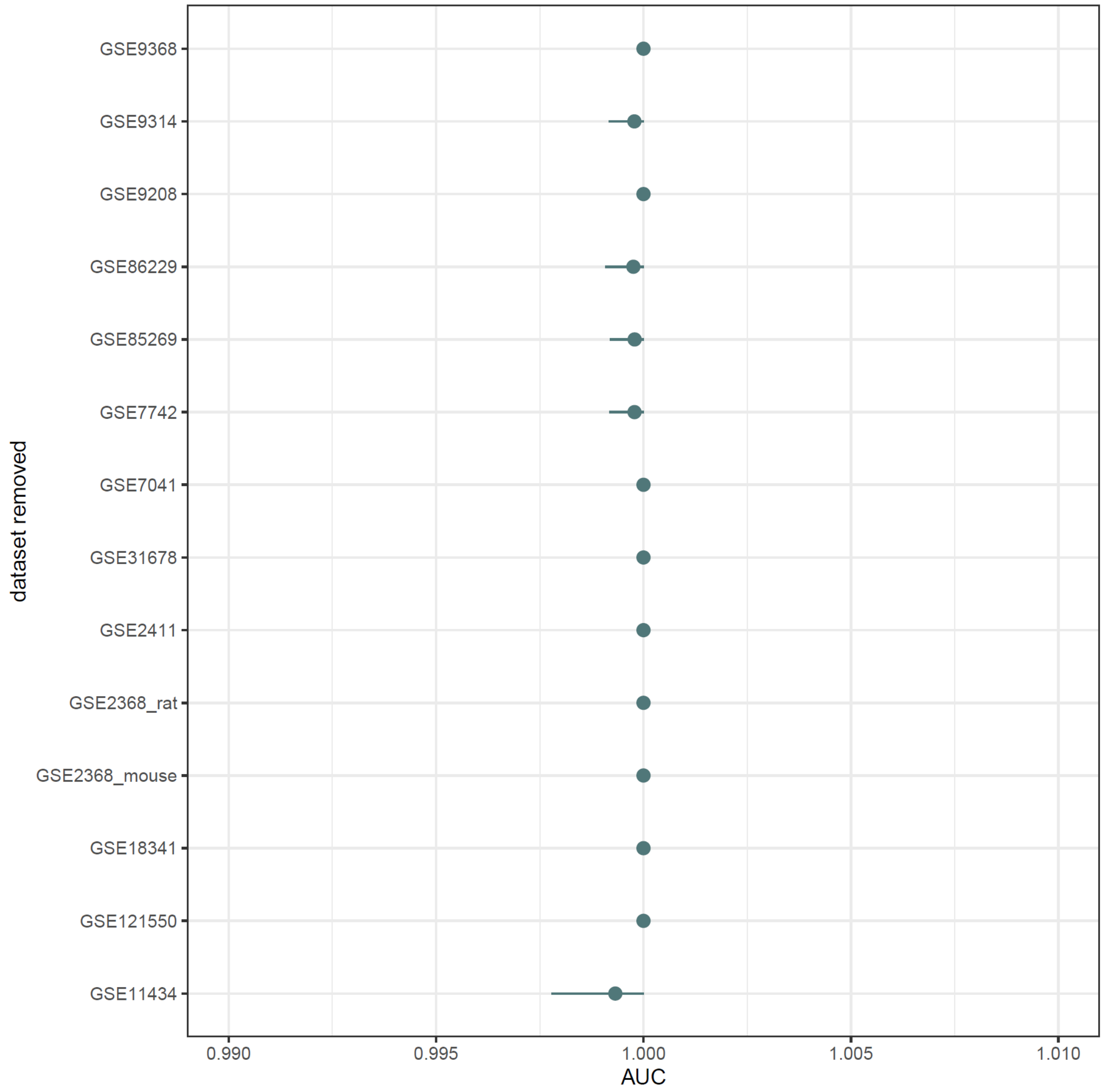

Figure S12. Distribution of area under the ROC curve (AUROC) of 10000 metascores generated using sets of 6 randomly chosen genes, to identify mechanically ventilated samples. The AUROCs are significantly lower than the AUROCs of the signatures obtained in the study.

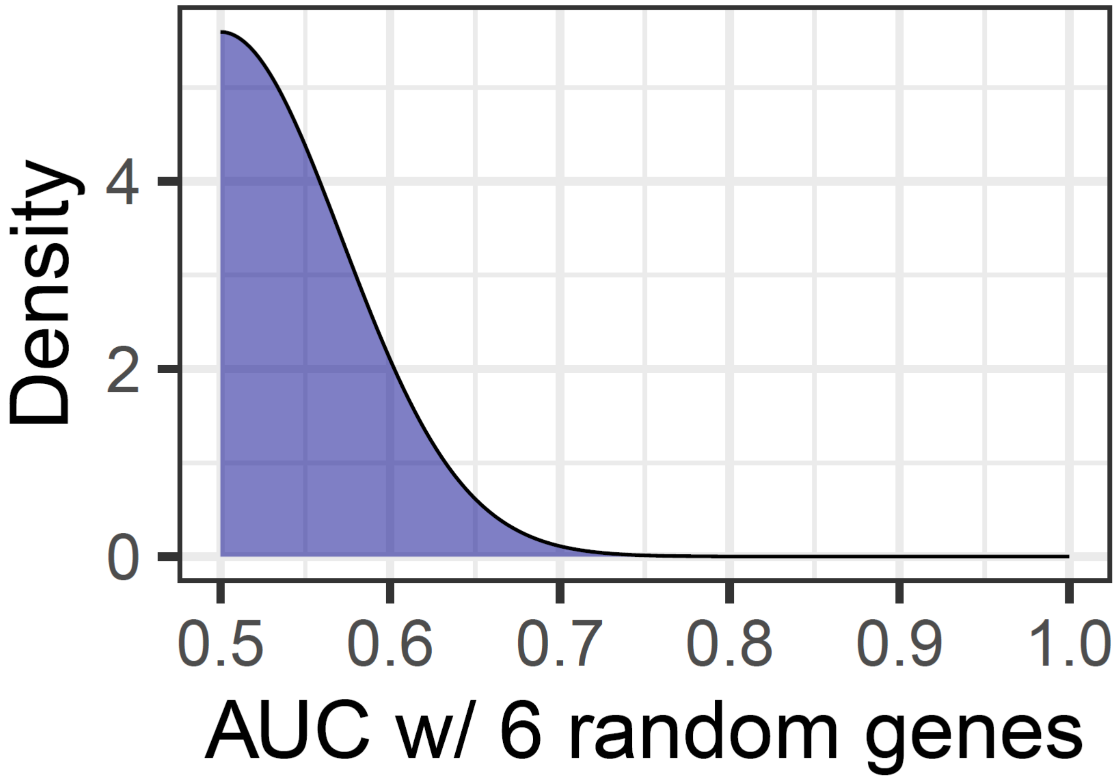

Figure S13. Expression of miRNAs and genes included in the described signatures in human lungs ventilated ex-vivo either with continuous positive airway pressure (CPAP) or with high tidal volumes to cause ventilator-induced lung injury (VILI).

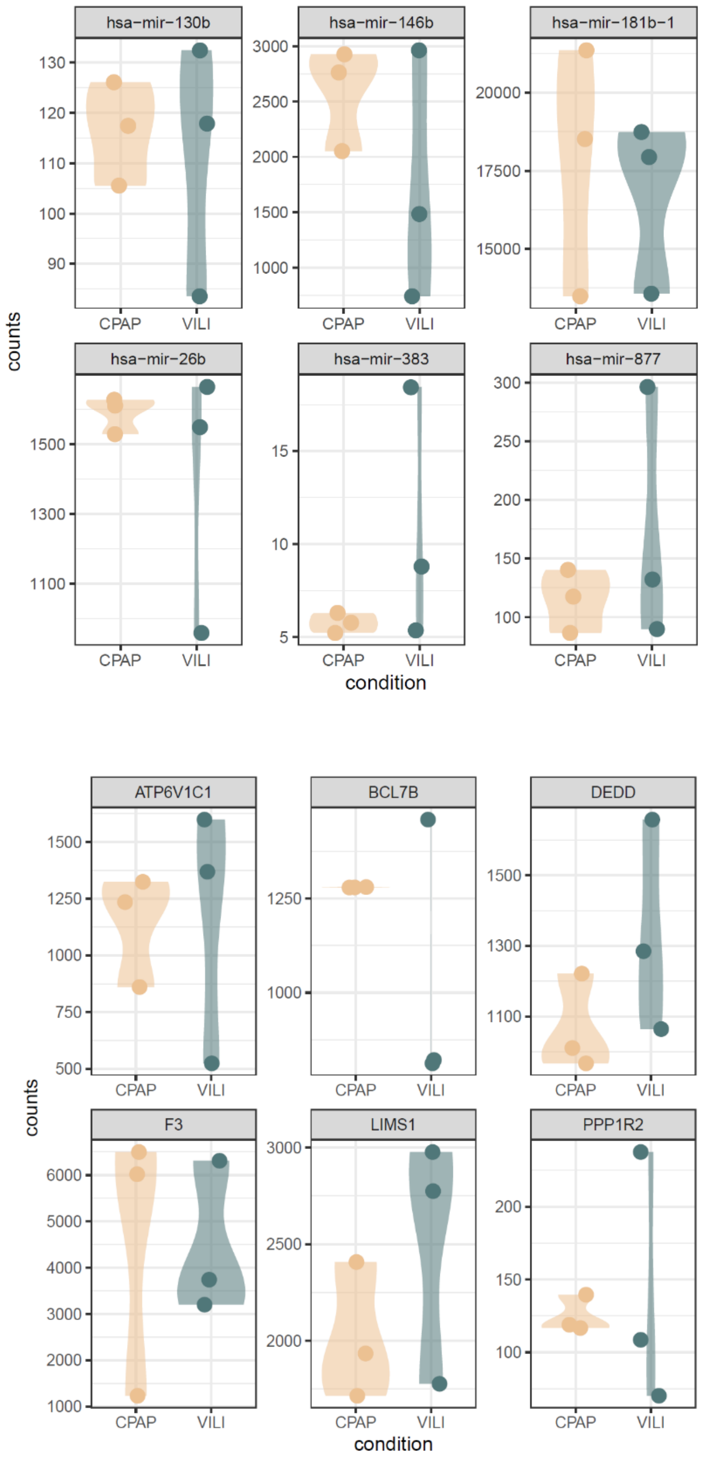

Figure S14. Changes in miRNAs abundance in bronchoalveolar lavage fluid from patients ventilated with different tidal volumes (6 or 3 ml/Kg of predicted body weight [PBW]) and stratified by their response in terms of lung strain, as a marker of lung overdistension.

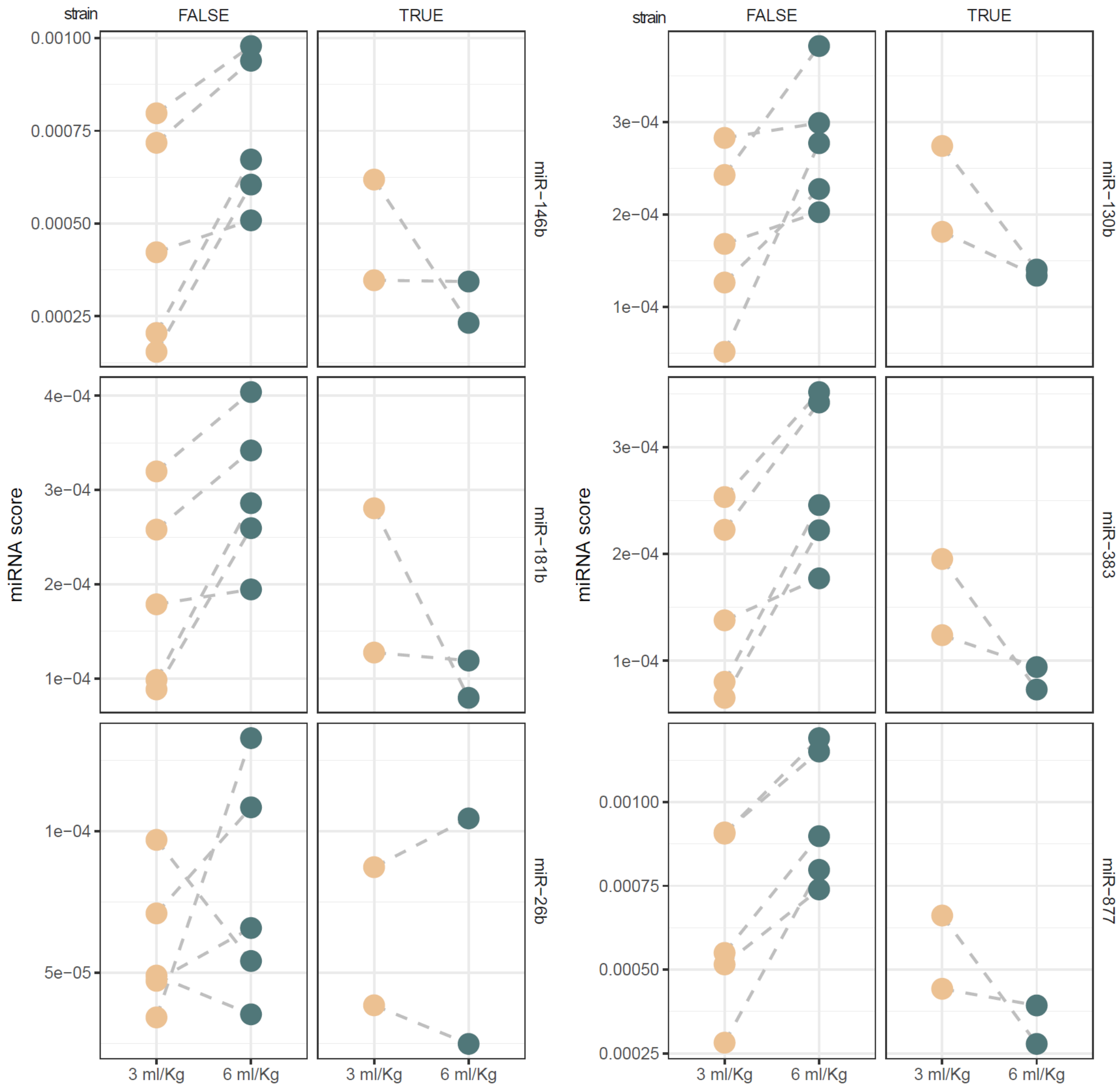

References

1. Sweeney TE, Wong HR, Khatri P. Robust classification of bacterial and viral infections via integrated host gene expression diagnostics. *Sci Transl Med* 2016; 8: 346ra91.
2. Wu T, Hu E, Xu S, Chen M, Guo P, Dai Z, Feng T, Zhou L, Tang W, Zhan L, Fu X, Liu S, Bo X, Yu G. clusterProfiler 4.0: A universal enrichment tool for interpreting omics data. *Innovation (N Y)* 2021; 2: 100141.
3. Xiao F, Zuo Z, Cai G, Kang S, Gao X, Li T. miRecords: an integrated resource for microRNA-target interactions. *Nucleic Acids Res* 2009; 37: D105-110.
4. Huang H-Y, Lin Y-C-D, Cui S, Huang Y, Tang Y, Xu J, Bao J, Li Y, Wen J, Zuo H, Wang W, Li J, Ni J, Ruan Y, Li L, Chen Y, Xie Y, Zhu Z, Cai X, Chen X, Yao L, Chen Y, Luo Y, LuXu S, Luo M, Chiu C-M, Ma K, Zhu L, Cheng G-J, Bai C, et al. miRTarBase update 2022: an informative resource for experimentally validated miRNA-target interactions. *Nucleic Acids Res* 2022; 50: D222–D230.
5. Karagkouni D, Paraskevopoulou MD, Chatzopoulos S, Vlachos IS, Tastsoglou S, Kanellos I, Papadimitriou D, Kavakiotis I, Maniou S, Skoufos G, Vergoulis T, Dalamagas T, Hatzigeorgiou AG. DIANA-TarBase v8: a decade-long collection of experimentally supported miRNA-gene interactions. *Nucleic Acids Res* 2018; 46: D239–D245.
6. Ru Y, Kechris KJ, Tabakoff B, Hoffman P, Radcliffe RA, Bowler R, Mahaffey S, Rossi S, Calin GA, Bemis L, Theodorescu D. The multiMiR R package and database: integration of microRNA-target interactions along with their disease and drug associations. *Nucleic Acids Res* 2014; 42: e133.
7. Dumigan A, Fitzgerald M, Santos JS-PG, Hamid U, O'Kane CM, McAuley DF, Bengoechea JA. A Porcine Ex Vivo Lung Perfusion Model To Investigate Bacterial Pathogenesis. *mBio* [Internet] 2019 [cited 2021 Apr 11]; 10Available from: https://www.ncbi.nlm.nih.gov/pmc/articles/PMC6890995/.
8. McAuley DF, Curley GF, Hamid UI, Laffey JG, Abbott J, McKenna DH, Fang X, Matthay MA, Lee JW. Clinical grade allogeneic human mesenchymal stem cells restore alveolar fluid clearance in human lungs rejected for transplantation. *Am J Physiol Lung Cell Mol Physiol* 2014; 306: L809-815.
9. Patro R, Duggal G, Love MI, Irizarry RA, Kingsford C. Salmon provides fast and bias-aware quantification of transcript expression. *Nat Methods* 2017; 14: 417–419.
10. Love MI, Huber W, Anders S. Moderated estimation of fold change and dispersion for RNA-seq data with DESeq2. *Genome Biol* 2014; 15: 550.
11. Friedländer MR, Mackowiak SD, Li N, Chen W, Rajewsky N. miRDeep2 accurately identifies known and hundreds of novel microRNA genes in seven animal clades. *Nucleic Acids Res* 2012; 40: 37–52.
12. Amado-Rodríguez L, Del Busto C, López-Alonso I, Parra D, Mayordomo-Colunga J, Arias-Guillén M, Albillos-Almaraz R, Martín-Vicente P, López-Martínez C, Huidobro C, Camporota L, Slutsky AS, Albaiceta GM. Biotrauma during ultra-low tidal volume ventilation and venoarterial extracorporeal membrane oxygenation in cardiogenic shock: a randomized crossover clinical trial. *Ann Intensive Care* 2021; 11: 132.
13. López-Martínez C, Martín-Vicente P, Gómez de Oña J, López-Alonso I, Gil-Peña H, Cuesta-Llavona E, Fernández-Rodríguez M, Crespo I, Salgado Del Riego E, Rodríguez-García R, Parra D, Fernández J, Rodríguez-Carrio J, Jimeno-Demuth FJ, Dávalos A, Chapado LA, Coto E, Albaiceta GM, Amado-Rodríguez L. Transcriptomic clustering of critically ill COVID-19 patients. *Eur Respir J* 2023; 61: 2200592.

14. Carvalho BS, Irizarry RA. A Framework for Oligonucleotide Microarray Preprocessing. *Bioinformatics* Oxford, UK: Oxford University Press; 2010; 26: 2363–2367.
15. Ritchie ME, Phipson B, Wu D, Hu Y, Law CW, Shi W, Smyth GK. limma powers differential expression analyses for RNA-sequencing and microarray studies. *Nucleic Acids Research* 2015; 43: e47.
16. Haynes WA, Vallania F, Liu C, Bongen E, Tomczak A, Andres-Terrè M, Lofgren S, Tam A, Deisseroth CA, Li MD, Sweeney TE, Khatri P. Empowering multi-cohort gene expression analysis to increase reproducibility. *Pac Symp Biocomput* 2017; 22: 144–153.
17. Wickham H. ggplot2: Elegant Graphics for Data Analysis [Internet]. Springer-Verlag New York; 2016.Available from: https://ggplot2.tidyverse.org.
18. Robin X, Turck N, Hainard A, Tiberti N, Lisacek F, Sanchez J-C, Müller M. pROC: an open-source package for R and S+ to analyze and compare ROC curves. *BMC Bioinformatics* 2011; 12: 77.
